# *Salmonella enterica *serovar Typhimurium adhesion and cytotoxicity during epithelial cell stress is reduced by *Lactobacillus rhamnosus *GG

**DOI:** 10.1186/1757-4749-1-14

**Published:** 2009-07-09

**Authors:** Kristin M Burkholder, Arun K Bhunia

**Affiliations:** 1 Molecular Food Microbiology Laboratory, Department of Food Science, 745 Agriculture Mall Drive, Purdue University, West Lafayette, Indiana 47907-2009, USA

## Abstract

**Background:**

Physiological stressors may alter susceptibility of the host intestinal epithelium to infection by enteric pathogens. In the current study, cytotoxic effect, adhesion and invasion of *Salmonella enterica *serovar Typhimurium (*S*. Typhimurium) to Caco-2 cells exposed to thermal stress (41°C, 1 h) was investigated. Probiotic bacteria have been shown to reduce interaction of pathogens with the epithelium under non-stress conditions and may have a significant effect on epithelial viability during infection; however, probiotic effect on pathogen interaction with epithelial cells under physiological stress is not known. Therefore, we investigated the influence of *Lactobacillus rhamnosus *GG and *Lactobacillus gasseri *on *Salmonella *adhesion and *Salmonella*-induced cytotoxicity of Caco-2 cells subjected to thermal stress.

**Results:**

Thermal stress increased the cytotoxic effect of both *S*. Typhimurium (P = 0.0001) and nonpathogenic *E. coli *K12 (P = 0.004) to Caco-2 cells, and resulted in greater susceptibility of cell monolayers to *S*. Typhimurium adhesion (P = 0.001). Thermal stress had no significant impact on inflammatory cytokines released by Caco-2 cells, although exposure to *S*. Typhimurium resulted in greater than 80% increase in production of IL-6 and IL-8. Blocking *S*. Typhimurium with anti-ShdA antibody prior to exposure of *Salmonella *decreased adhesion (P = 0.01) to non-stressed and thermal-stressed Caco-2 cells. Pre-exposure of Caco-2 cells to *L. rhamnosus *GG significantly reduced *Salmonella*-induced cytotoxicity (P = 0.001) and *Salmonella *adhesion (P = 0.001) to Caco-2 cells during thermal stress, while *L. gasseri *had no effect.

**Conclusion:**

Results suggest that thermal stress increases susceptibility of intestinal epithelial Caco-2 cells to *Salmonella *adhesion, and increases the cytotoxic effect of *Salmonella *during infection. Use of *L. rhamnosus *GG as a probiotic may reduce the severity of infection during epithelial cell stress. Mechanisms by which thermal stress increases susceptibility to *S*. Typhimurium colonization and by which *L. rhamnosus *GG limits the severity of infection remain to be elucidated.

## Background

*Salmonella enterica *are important facultative intracellular pathogens that cause gastroenteritis in humans [1]. The diverse *Salmonella *genus contains over 2500 serotypes [2], all of which are potentially pathogenic to humans [3]. Specifically, *Salmonella enterica *serovar Typhimurium (*S*. Typhimurium) is implicated in human foodborne illnesses and often enters the human food supply via contamination of poultry, pork, beef and dairy products, and nuts such as peanuts and pistachios. In recent years, antibiotic-resistant strains of *Salmonella *have emerged, and salmonellosis caused by multi-drug resistant *S*. Newport and *S*. Typhimurium DT104 has caused great public health concern [4-6].

Adhesion of *Salmonella *to the intestinal epithelial surface is a key first step in pathogenesis and is central to its colonization of the intestine [7]. Although *Salmonella *can colonize a healthy host, the greatest risk for intestinal infection by enteric pathogens may occur during periods of physiological stress. *In vivo *animal studies have shown that during periods of stress, susceptibility to *Salmonella *colonization and infection increases [8-10]. Less is known of how physiological stress may influence the interaction of *Salmonella *with the human intestinal epithelium, although it is likely to follow a similar trend as other animal models. Proposed mechanisms for increased *Salmonella *colonization during stress have largely focused on the deleterious effect of stress on host immunity. While impaired immune function certainly contributes to stress-related infection, little information exists about the influence of stress on susceptibility of epithelial cells to infection. The intestinal epithelium is a crucial, but thin, barrier, which is susceptible to perturbation by stress [11,12]. In particular, epithelial damage elicited by stress may expose or cause apical secretion of extracellular matrix proteins such as fibronectin [13], which serves as receptor for the MisL and ShdA adhesion proteins of *S*. Typhimurium [14,15].

Recent research has indicated that most physiological and psychological stressors have an impact on gut health and susceptibility to enteric pathogens [16]. For the current study, we used high temperature (41°C, 1 h) to examine the influence of stress on *Salmonella *colonization of cultured intestinal epithelial cells and the effectiveness of probiotic Lactobacilli for altering the outcome of infection. Core temperatures ranging from 39°C to 42.5°C are physiologically relevant and have been reported in humans suffering from mild to severe thermal stress [12]. The deleterious effects of thermal stress are first manifested in the gut [12] in humans and animals, and we and others have shown that high temperature stress damages the intestinal epithelium using animal and cell culture models [11,17-19]. Therefore, we chose to use thermal stress as a model physiological stressor that, like other physiological or psychological stressors, can alter normal intestinal homeostasis and may influence the outcome of infection. Specifically, stress due to high temperature (39°C – 42°C) can elicit enterocyte membrane damage or death [11,18], alter of normal villus/crypt structure [17,18], impair tight junction integrity [20], and may enhance susceptibility to colonization and infection by enteric microorganisms. Greater vulnerability to enteric microbes is demonstrated by increased LPS concentration in the blood following thermal stress [19,21], and indicates that a compromised gut barrier may lead to opportunistic infection. However, little information exists on the influence of thermal stress on epithelial susceptibility to pathogen binding and cytotoxicity.

Inhibition of pathogen adhesion to the intestinal epithelium may prevent colonization and limit opportunity for systemic infection [8,22]. Certain probiotic bacteria, including *Lactobacillus *and *Bifidobacteria*, may be effective in ameliorating negative intestinal effects of stress [23,24] or preventing adhesion and invasion by enteric pathogens, including Salmonellae [25-29]. Although the exact mechanism of action is unknown, probiotics could reduce intestinal infections by competing with pathogens for binding sites on the intestinal wall, competing for nutrients within the intestinal lumen, producing antibacterial compounds or lactic acid, or by stimulating the host immune system [30-34]. Despite numerous studies which demonstrate antipathogenic properties of probiotics, great variability exists in their reported effectiveness in reducing intestinal infection [23,35] and this variability may depend on which probiotic organism is used, as well as health status of the host. In fact, recent studies suggest that probiotics may be most effective when normal intestinal homeostasis is perturbed, particularly during periods of stress [16]. Therefore, in the current study, we examined the influence of stress on colonization of *S*. Typhimurium and epithelial membrane damage, and determined the influence of *L. rhamnosus *GG and *L. gasseri *on the *Salmonella*-epithelial interaction.

## Results

### Influence of thermal stress on bacterial adhesion, invasion, and cytotoxic effects

**Figure 1:**
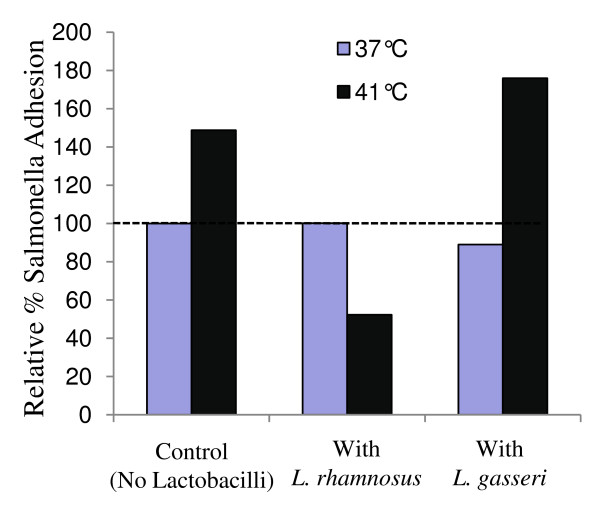
**Effect of pre-exposure of *Lactobacillus rhamnosus *GG or *L. gasseri *to thermal-stressed (41°C, 1 h) Caco-2 cells on adhesion of *Salmonella enterica *serovar Typhimurium**. Percent adhesion values are given relative to the adhesion of *S*. Typhimurium to Caco-2 cells at 37°C without exposure to Lactobacilli, which were taken as 100%. The percent error rates for treatments are as follows: Control (no Lactobacilli) at 37°C – 2.12%, Control (no Lactobacilli) at 41°C – 1.89%, with *L. rhamnosus *at 37°C – 2.9%, with *L. rhamnosus *at 41°C – 4.43%, with *L. gasseri *at 37°C – 7.2%, with *L. gasseri *at 41°C – 10.8%.

*In vitro *cell culture experiments were conducted to determine the effect of thermal stress on cellular susceptibility to bacterial attachment, invasion and on lactate dehydrogenase (LDH) release (% cytotoxicity) from monolayers exposed to bacteria. Subjecting Caco-2 cells to thermal stress (41°C) for 1 h prior to bacterial exposure increased adhesion of *S*. Typhimurium by more than 1.7-fold (P = 0.001), whereas adhesion of nonpathogenic *E. coli *K12 was unchanged (Table 1, Fig 1). Bacterial invasion of Caco-2 cells was not influenced by thermal stress (Table 1, Fig 1). Greater adhesion of nonpathogenic *E. coli *K12 than *S*. Typhimurium was observed only at 37°C, and is likely due to expression of pili and other adhesion molecules which enable intestinal commensal organisms to colonize without causing infection [36].

**Table 1:** Adhesion, invasion and cytotoxic effect of *Salmonella enterica *serovar Typhimurium and *E. coli *K12 on thermal-stressed (41°C for 1 h) Caco-2 cells

**Bacteria**	**% Adhesion**		**% Invasion**		**% Cytotoxicity**	
	
	37°C	41°C	37°C	41°C	37°C	41°C
*S*. Typhimurium	5.63 ± 1.19^bB^	16.39 ± 5.68^aA^	0.09 ± 0.01^A^	0.08 ± 0.01^A^	8.32 ± 1.36^bA^	30.52 ± 1.08^aA^
*E. coli *K12	13.40 ± 1.07^A^	15.10 ± 1.45^A^	0.003 ± 0^B^	0.003 ± 0^B^	8.04 ± 1.43^bA^	20.68 ± 1.43^aB^
*L. rhamnosus *GG	1.82 ± 0.12^b^	5.28 ± 0.49^a^	NT	NT	5.31 ± 0.96^B^	7.32 ± 4.6^C^
*L. gasseri*	4.47 ± 1.13^a^	1.58 ± 0.34^b^	NT	NT	6.05 ± 0.5^B^	5.52 ± 1.14^C^
No bacteria	NT	NT	NT	NT	0^bC^	9.46 ± 1.08^aD^

Values represent 12 wells per treatment. Rows labeled with (a, b) and columns labeled with (A, B, C) bearing different superscripts within events are significantly different according to Duncan's test (P < 0.05). NT, not tested

**Figure 2:**
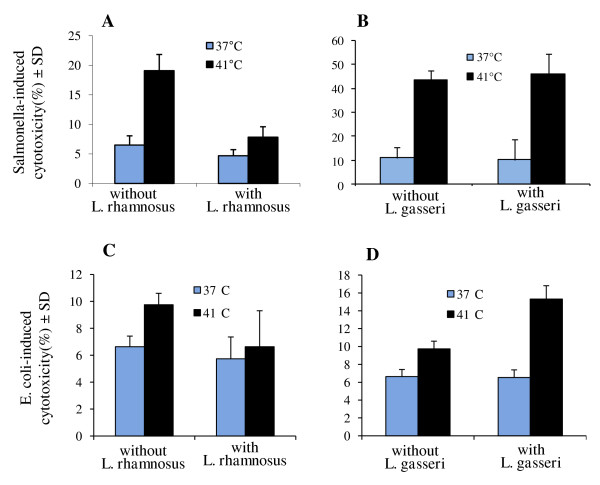
**Effect of pre-exposure of (A, C) *Lactobacillus rhamnosus *GG, (B, D) *L. gasseri *to thermal-stressed (41°C, 1 h) or untreated (37°C) Caco-2 cells on cytotoxicity induced by *Salmonella enterica *serovar Typhimurium or *E. coli *K12**.

LDH release (percent cytotoxicity) increased by over 3.5-fold (P = 0.0001) when Caco-2 cells were subjected to both thermal stress and *S*. Typhimurium infection compared to unstressed, uninfected cells (Table 1). Exposure to *E. coli *K12 also induced cytotoxicity in thermally-stressed cells (P = 0.0004), albeit at a lower level than observed with *S*. Typhimurium. Since high temperature stress in absence of bacteria induced a more modest increase (P = 0.005) in LDH release (Table 1, Fig 2), it is likely that exposure of stressed Caco-2 cells to bacterial products led to induction of the cytotoxic response. Interestingly, exposure to *L. rhamnosus *or *L. gasseri *did not induce a significant change in LDH release at either temperature, suggesting that bacteria-induced cytotoxicity observed with *Salmonella *and *E. coli *may be specifically due to Gram-negative microbial products (Table 1).

### Adhesion and cytotoxic effect of S. Typhimurium on thermal-stressed Caco-2 cells pre-exposed to Lactobacilli

We sought to determine the influence of probiotics *L. rhamnosus *and *L. gasseri *on adhesion and cytotoxic effect of *Salmonella *to intestinal cell lines under normal and stress conditions by exposing Caco-2 monolayers to *L. rhamnosus *or *L. gasseri *for 1 h prior to stress and/or infection. *L. rhamnosus *did not alter adhesion of *Salmonella *to unstressed cells, but a significant reduction (P = 0.001) in *Salmonella *adhesion was observed in thermal-stressed cells that had been pre-exposed to *L. rhamnosus *(Fig 1). Similarly, *L. rhamnosus *offered a protective effect against cytotoxicity induced by *Salmonella *and *E. coli *K12 in-thermal stressed Caco-2 monolayers, reducing cytotoxicity by over 3-fold and 1.5-fold, respectively (Fig 2). In contrast, *L. gasseri*, another probiotic strain [37], did not protect Caco-2 cells from *Salmonella *adhesion (Fig 1) or *Salmonella *or *E. coli*-induced cytotoxicity (Fig 2). The differences in protection offered by *L. rhamnosus *and *L. gasseri *against *Salmonella *adhesion and bacterial-induced cytotoxicity may be due to their own relative adhesion levels during thermal stress; at 41°C, adhesion of *L. rhamnosus *GG increased (P = 0.03), whereas adhesion of *L. gasseri *decreased (P = 0.02) (Table 1).

### Adhesion of S. Tyhpimurium after blocking bacterial surface with anti-ShdA antibody

**Figure 3:**
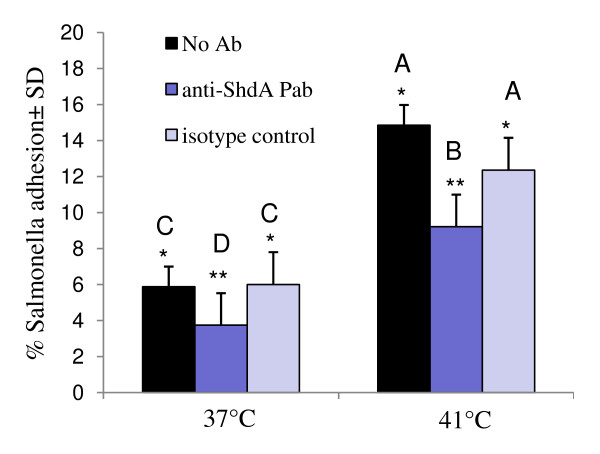
**Influence of anti-ShdA antibody in adhesion of *S*. Typhimurium to unstressed (37°C) or thermal-stressed (41°C, 1 h) Caco-2 cells**. Prior to infection of Caco-2 cell monolayers, *S*. Typhimurium cells were treated with anti-ShdA antibody, isotype control anti-*Salmonella *polyclonal antibody or left untreated. Bars marked with different letters (A, B, C, D) are significantly different at P < 0.05.

Since *S*. Typhimurium ShdA aids in epithelial adhesion by binding to host fibronectin [14], we tested the influence of ShdA in adhesion during normal and stress conditions, when epithelial fibronectin may be more available. Blocking of *Salmonella *ShdA with anti-ShdA antibody prior to infection of Caco-2 cells resulted in a reduction in *Salmonella *binding from 5.9 to 3.7% (P = 0.01) in unstressed cells and from 14.9% to 9.2% (P = 0.003) in thermal-stressed monolayers (fig 3).

### Transmission electron microscopy of thermal-stressed Caco-2 cells infected with S. Typhimurium

**Figure 4:**
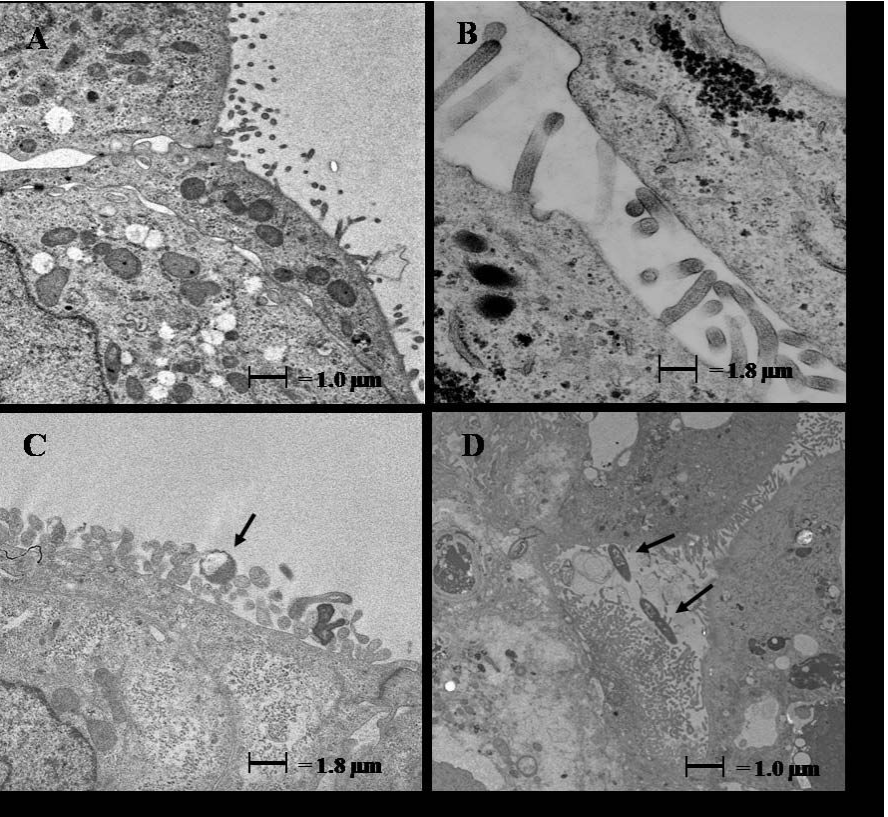
**Transmission electron micrograph of Caco-2 cells following thermal stress and *Salmonella enterica *serovar Typhimurium exposure**. (A) Caco-2 cells with no heat treatment and no added bacteria, (B) Caco-2 cells after thermal stress (41°C, 1 h), (C) non thermal-stressed Caco-2 cells exposed to *S*. Typhimurium (10^6 ^cfu/ml for 1 h), and (D) thermal-stressed Caco-2 cells exposed to *S*. Typhimurium (10^6 ^cfu/ml for 1 h). Arrows indicate *S*. Typhimurium cells.

TEM was used to visualize the Caco-2 cell surface architecture before and after thermal stress, as well as during the interaction between *S*. Typhimurium and the thermal-stressed Caco-2 cell surface. In Caco-2 cells with no bacterial exposure (37°C and 41°C treatments), cells exhibited microvilli and surface projections characteristic of actively growing cells undergoing pinocytosis (Fig 4A, B) [38], indicating that the thermal stress conditions were not sufficient to alter cell surface morphology. In unstressed Caco-2 cells, *S*. Typhimurium were observed in close proximity to the eukaryotic cells (Fig 4C), but more bacteria were seen in contact with the Caco-2 cells when cells had been subjected to high temperature (Fig 4D).

### Influence of thermal stress and S. Typhimurium infection on cytokine expression in Caco-2 cells

**Figure 5:**
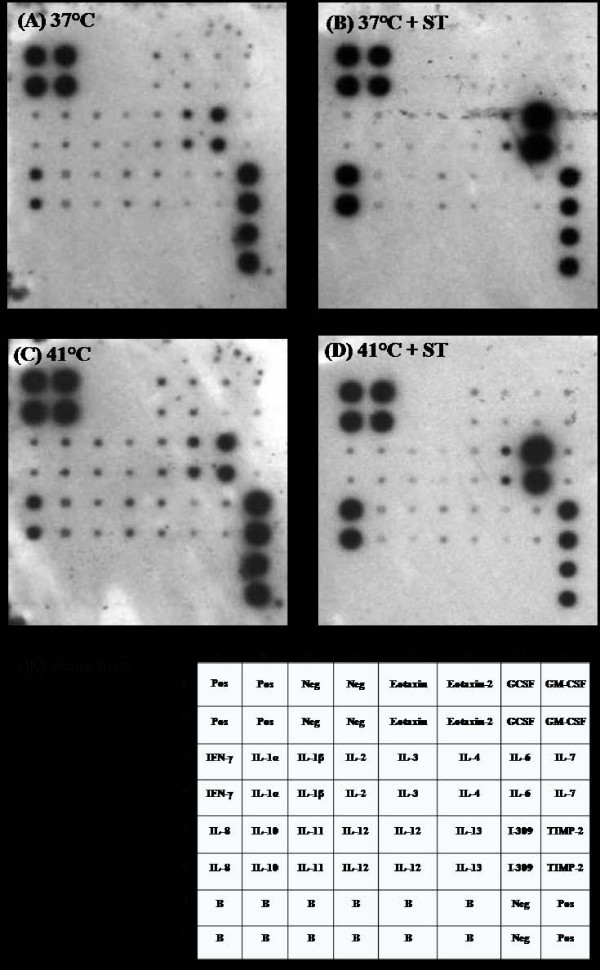
**Immunoblot cytokine array analysis of Caco-2 cells held at (A) 37°C, or exposed to (B) *S*. Typhimurium (ST) for 1 h at 37°C, (C) 41°C thermal stress for 1 h, or (D) 41°C thermal stress for 1 h followed by 1 h of *S*. Typhimurium exposure**. (E) Template showing the position of different cytokines. Pos, positive control; Neg, negative control; B, blank; GCSF, granulocyte colony stimulating factor; GM-CSF, granulocyte monocyte-colony stimulating factor; I-309, a CC chemokine; TIMP-2, tissue inhibitor of metalloproteinase-2.

Since host cytokines can affect intestinal homeostasis [39,40], we determined the influence of thermal stress and *S*. Typhimurium infection on expression of cytokines by Caco-2 cells. As reported by others, *S*. Typhimurium infection induced greater than 80% increase in expression of IL-6 and IL-8 [41,42], but subjecting Caco-2 cells to thermal stress alone (41°C, 1 h) did not alter cytokine expression (Fig 5).

## Discussion

Stress, whether physical or psychological, can have a notable effect on host physiology, with the earliest and greatest impact occurring in the gastrointestinal tract [43]. *In vitro *work has shown deleterious effects of stress on intestinal integrity [16,18], which may enhance pathogen adherence to the intestinal epithelium. Interestingly, some reports show that multiple exposures to mild stress can induce a cytoprotective effect in intestinal epithelial cells against future, more severe, stressors, likely due to induction of the heat shock response [44]. However, *in vitro *models have shown that acute stress can decrease transepithelial resistance of epithelial cells [43,45], increase expression or secretion of proteins such as fibronectin [13] or heat shock proteins [46,47], which are targeted as receptors by some enteric pathogens [13,48,49]. While *in vivo *studies with food producing animals have associated stress with increased intestinal colonization and shedding of *Salmonella *and other enteric pathogens [9,10], less is known of how stress may influence *Salmonella *interaction with the human intestinal epithelium, or how probiotic bacteria may mediate this interaction.

Here we report increased binding of *S*. Typhimurium to Caco-2 cells following 1 h of thermal stress (41°C) (Table 1, Fig 1). Epithelial cells may be subjected to stress in a variety of ways, and it is worth noting that bacterial infection itself may serve as a stressor to host tissues. A natural *S*. Typhimurium infection can induce fever in humans and animals [50,51]. Little information exists on the influence of fever on epithelial colonization by *Salmonella*, and although many systemic host factors are involved in the fever response that cannot be accounted for in a cell culture model, our data suggests that high temperature may influence epithelial susceptibility for infection.

Thermal stress alone elicited a mild increase in LDH release (9.46% cytotoxicity) from Caco-2 cells, agreeing with previous reports of epithelial cell damage induced by temperatures near 41°C [52]. However, transmission electron micrographs showed that this heat treatment alone was not sufficient to cause discernable changes in epithelial structure (Fig 4A, B). However, LDH release was greatest when stressed cells were infected with *S*. Typhimurium and to a lesser extent following exposure to nonpathogenic *E. coli *K12 (Table 1). The high level of cytotoxicity observed during infection with *S*. Typhimurium was likely due to membrane damage elicited by enterotoxins [53] (Table 1). Exposure of Caco-2 cells to *L. rhamnosus *or *L. gasseri *did not induce LDH release, suggesting that Gram-negative bacterial products, absent in Lactobacilli, may have enhanced the cytotoxic effect of high temperature. Interestingly, others have reported potential cytoprotective effects of Lactobacilli on intestinal epithelial cells [54,55], and exposure to these probiotics may protect the epithelium against negative effects of physiological stress or infection [38,56].

We also tested the influence of high temperature and *Salmonella *exposure on cytokine expression in Caco-2 cells, since certain cytokines can alter gut integrity and influence the outcome of infection [32,39,40]. As reported by others, *Salmonella *infection significantly increased expression of IL-6 and IL-8 [41,42]. However, we found no effect of thermal stress (Fig 5) on cytokine levels, indicating that alteration of epithelial cytokine production is not a likely mechanism by which stress affects intestinal susceptibility to *S*. Typhimurium colonization.

Initial adhesion to the intestine is the critical first step in establishing colonization or infection of the host [7]. Recent studies have demonstrated the importance of genes encoded on SPI3 in intestinal colonization of *S*. Typhimurium. SPI3-encoded T5SS (Type 5 Secretion System) pathway members MisL and ShdA were shown to bind to intestinal fibronectin in the mouse and mediate persistent *S*. Typhimurium colonization [14,15]. Disturbance of the intact epithelial barrier by stress or disease may increase exposure of basolateral proteins such as fibronectin, and may increase opportunity for pathogen binding. In the current study, we used a ShdA-specific antibody to block the ShdA protein on the surface of *S*. Typhimurium prior to conducting adhesion assays. The preliminary data showed that blocking ShdA reduced *S*. Typhimurium adhesion to normal and stressed Caco-2 cells, confirming that ShdA is also important for binding to human intestinal cells. While treating *Salmonella *with the anti-ShdA antibody did significantly reduce adhesion to thermal-stressed monolayers, adhesion after antibody treatment was still greater than that observed in unstressed cells. This indicates that while fibronectin exposure may play an important role in *Salmonella *colonization during stress and non-stress conditions, it is not the only factor involved in promoting colonization during epithelial cell stress. Indeed, a variety of pili and adhesion molecules also contribute to *Salmonella *binding and invasion during normal host condition [57] and are likely to promote binding when intestinal homeostasis is perturbed [58].

Previous reports have demonstrated the ability of probiotic bacteria to decrease pathogen binding and ameliorate mucosal damage elicited by infection. We recently showed that *Lactobacillus bulgaricus *inhibits binding and cytotoxic effect of *Clostridium difficile *with a Caco-2 cell model [59]. In addition, probiotics *Streptococcus thermophilus *and *Lactobacillus acidophilus *limited adhesion and invasion of enteroinvasive *E. coli*, and increased transepithelial resistance and tight junction integrity during infection [28]. Exposing epithelial cells to *Lactobacillus casei *prior to infection with adherent-invasive *E. coli *reduced adhesion of the pathogen by 73% [25].

In the current study, we examined the influence of *L. rhamnosus *GG and *L. gasseri *on *Salmonella *infection during acute epithelial stress. We chose these organisms because numerous reports indicate their effectiveness as probiotics, by improving epithelial integrity during infection [60,61], and by limiting pathogen binding through either direct competition or by lactic acid production [38,62]. Here, we demonstrate that *L. rhamnosus *GG significantly reduced the cytotoxic effect of *Salmonella *in thermal-stressed Caco-2 cells, which agrees with other reports of the effectiveness of this probiotic in improving mucosal integrity and epithelial cell health during infection or exposure to toxins [60,63]. We also observed that *L. rhamnosus *significantly decreased *Salmonella *adhesion to stressed Caco-2 cells, but did not alter binding to unstressed cells (Fig 1C). Unlike *L. rhamnosus*, *L. gasseri *neither protected Caco-2 cells from the cytotoxic effect of high temperature and *S*. Typhimurium, nor altered adhesion of *Salmonella*. In contrast to our data, others found that both *L. rhamnosus *GG and *L. gasseri *limited adhesion of *Salmonella *[61] and *E. coli *[38,64] to unstressed host cells. These discrepancies could be due to differences in the specific strains of *L. gasseri *or *L. rhamnosus *used in those studies, or to differences in the dose of probiotic or pathogen applied in the infection studies.

## Conclusion

All together, our data indicate that physiological stress can increase epithelial susceptibility to *S*. Typhimurium adhesion and *Salmonella*-induced cytotoxicity. We show that *L. rhamnosus *GG may serve to protect against *S*. Typhimurium infection during periods of stress, by using some unique mechanism which is not employed by *L. gasseri*. Future work will focus on understanding what specific changes occur in the host epithelium during acute stress that promotes *Salmonella *binding, and how *L. rhamnosus *GG limits this binding and reduces host cell damage during infection.

## Methods

### Bacterial strains and growth conditions

*Salmonella enterica *serovar Typhimurium Copenhagan and *Escherichia coli *K12 were from our culture collection. *E. coli *K12 was used as a Gram-negative, nonpathogenic control. *Lactobacillus rhamnosus *strain GG and *L. gasseri *1 SL4 were purchased from American Type Culture Collection (ATCC, Manassas, VA), and were indigenous human intestinal species that have been identified as potential probiotic organisms [38,60,65].

*S*. Typhimurium and *E. coli *K12 stock cultures were grown in Luria Bertani (LB) broth and stored at -20°C with the addition of 20% (vol/vol) glycerol. Cultures were grown statically overnight in LB broth at 37°C, transferred twice to fresh LB broth and grown overnight for the Caco-2 cell culture assays. Lactobacilli were grown in deMann-Rogosa-Sharpe (MRS) broth and stored at -20°C with the addition of 20% (vol/vol) glycerol. For active cultures, they were grown statically overnight in MRS broth at 37°C in microaerophilic atmosphere (7% CO_2_), transferred twice to fresh MRS broth and grown overnight for the cell culture assays. All bacterial cells were harvested by centrifugation (1,469 × g for 15 min at 4°C), then washed three times in 0.02 M sterile phosphate buffered saline, pH 7.2 (PBS) and resuspended to a concentration of approximately 1 × 10^8 ^cfu/ml in PBS.

### Cultured cell lines

The Caco-2 (HTB37) human colon adenocarcinoma cell line (ATCC, Manassas, VA) was routinely cultured in Dulbeccos' Modified Eagles' Medium (DMEM; Sigma) with 10% (vol/vol) fetal bovine serum (FBS; Sigma). Cells were seeded in 24-well tissue culture dishes (Corning Life Sciences, New York, USA) or on 12-mm etched glass coverslips (EM Sciences, Fort Washington, Pa.) and grown to confluence at 37°C under 7% CO_2_.

### Cytotoxicity Assay

A cytotoxicity assay was used to quantify cell damage that occurred due to thermal stress or in the presence of pathogenic or probiotic bacteria. Cytotoxicity was determined using the Cytotoxicity Detection Kit (Roche Applied Science; Indianapolis, IN) which measures lactate dehydrogenase (LDH) release from the cytosol of damaged Caco-2 cells into the supernatant [66,67]. Two controls were included for calculation of percent cytotoxicity. Low controls consisted of supernatant from non-stressed Caco-2 cells with no exposure to bacteria. High controls were from cells treated with 1% Triton X-100 for one minute. All supernatants were centrifuged (800 × g for 5 min) to remove bacterial and eukaryotic cells. A 100 μl aliquot of each sample was dispensed in triplicate wells of a 96 well plate and LDH activity was determined as per manufacturer's protocol (Roche).

### Adhesion, invasion and cytotoxicity analyses with thermal-stressed intestinal cell line

The influence of thermal stress on susceptibility of Caco-2 cells to bacterial attachment and cytotoxicity was evaluated. Wells containing confluent cell monolayers were washed three times with FBS-free DMEM and subjected to thermal stress (41°C for 1 h). After heat stress, Caco-2 cells were inoculated with washed bacterial suspensions (*S*. Typhimurium or *E. coli *K12) at a multiplicity of exposure (MOE) of about 100:1, and were incubated at 37°C or 41°C for 1 h in 7% CO_2 _atmosphere. Media from each well were removed and analyzed for LDH activity (% cytotoxicity) as above. To analyze bacterial attachment, the same wells containing Caco-2 cells were washed 5 times each with 1 ml of cell-PBS (137 mM NaCl, 5.4 mM KCl, 3.5 mM Na_2_HPO_4_, 4.4 mM NaH_2_PO_4_, 11 mM glucose, pH 7.2) to remove non-adherent bacterial cells [68]. Monolayers were treated with 0.1% Triton X-100 for 10 min, serially diluted and plated on LB agar for enumeration of *S*. Typhimurium and *E. coli *K12.

To quantify bacterial invasion, monolayers were first exposed to *Salmonella *for 1 h, washed and then treated with 1 ml of gentamicin (100 μg/ml) suspended in DMEM for 1 h. Media were removed, cells were washed 5 times with cell PBS, treated with 0.1% Triton X-100 for 10 min, serially diluted and plated on LB agar as above.

### Influence of pre-exposure to Lactobacilli on adhesion and cytotoxicity of S. Typhimurium to Caco-2 cells

The influence of *L. rhamnosus *GG and *L. gasseri *on susceptibility of Caco-2 cell line to *S*. Typhimurium attachment was determined under normal (37°C) and thermal stress (41°C) conditions. *L. rhamnosus *or *L. gasseri *were added at a MOE of 100:1 to Caco-2 cells at 37°C for 1 h. Caco-2 cells with lactobacilli were held at 37°C or 41°C for an additional hour, and a 0.1 ml aliquot of *S*. Typhimurium (4 × 10^6 ^cfu/ml) was added to wells with an MOE of about 4:1, and plates were incubated at 37°C or 41°C for an hour. Media were removed from experimental wells to analyze for LDH activity and adhesion of *Salmonella *was determined as above.

### Role of ShdA in adhesion of S. Typhimurium

Prior to addition of *S*. Typhimurium to Caco-2 cells, bacteria were incubated for 1 h with 1 μg/ml anti-ShdA PAb (donated by Professor Andreas Baumler) or an isotype control anti-Salmonella polyclonal antibody from our lab (unpublished). Bacteria were added to thermal-stressed (41°C, 1 h) or control (37°C) Caco-2 monolayers and incubated for 1 h. Nonadherent bacteria were removed by washing, and adherent bacteria were enumerated as described above.

### Cytokine expression in Caco-2 cells following thermal stress and infection

Influence of thermal stress and *S*. Typhimurium infection on Caco-2 cytokine expression was determined. Following exposure to thermal stress and/or *S*. Typhimurium infection, the profile of cytokines expressed by Caco-2 cells was examined using the Human Inflammatory Cytokine Antibody Array (Ray Biotech, Norcross, GA) as described by the manufacturer. Briefly, Caco-2 cells in 24-well plates were either subjected to heat treatment (41°C) for 1 h, *S*. Typhimurium exposure for 1 h, or heat treatment (41°C, 1 h) followed by *S*. Typhimurium exposure for 1 h as described above. Following thermal stress or bacterial exposure, all wells including non-treated controls were incubated for 3 h with DMEM containing gentamicin to kill extracellular bacteria, and to allow time for cytokine production and secretion, after which supernatants were collected and pooled. The 3 h time point was chosen after an initial range of 1 to 5 h [42] was tested to determine optimal time required for cytokine release from Caco-2 cells (data not shown). Supernatants were applied to membranes containing cytokine antibody array (Ray Biotech) for 1 h at room temperature, and detection was performed with horseradish peroxidase-coupled secondary antibodies and chemiluminescence substrate (Ray Biotech). Array spot densities were determined and compared using Quantity One software (Bio-Rad, Hercules, CA).

### Transmission electron microscopy analysis of cell damage, bacterial binding and invasion

Transmission electron microscopy (TEM) was performed as described by van Tuinen and Riezman [69]. Caco-2 cells were subjected to 1 h of heat stress (41°C) and 1 h of *S*. Typhimurium exposure as described above. Media were removed and cells were washed 5 times with PBS. Cells were fixed in 3% paraformaldehyde containing 0.5% glutaraldehyde in PBS for 2 h at 4°C, washed three times in PBS, incubated in 1% sodium metaperiodate at room temperature for 1 h, and incubated in 50 mM ammonium chloride for 30 min. Pellets were embedded in 1.5% agarose and sliced into blocks that were dried in graded ethanol prior to embedding in LR White resin (EM Sciences, Fort Washington, PA). Cells were oven dried to polymerize the resins, which were thin-sectioned and placed on Formvar-coated nickel grids. The grids were air-dried and stained with 2% aqueous uranyl acetate (Sigma) for 3 min, washed again, and viewed under a transmission electron microscope (EM-400; Philips, Hillsboro, OR).

### Statistical analysis

Each adhesion, invasion and cytotoxicity experiment was repeated on three separate plates, with four replications per plate (12 total replications per treatment). Counts from adhesion and invasion assays, and cytotoxicity values were analyzed using the Generalized Linear Model (GLM) procedure of SAS, and significant differences were determined according to Duncan's test (SAS institute, Cary, NC). Data with a P value less than 0.05 was considered statistically significant.

## Competing interests

The authors declare that they have no competing interests.

## Authors' contributions

KB and AKB designed the study. KB carried out the experiments. KB and AKB wrote the manuscript and approved the final manuscript.
